# Different patterns of tibial plateau fractures associated with hyperextension injuries of the knee with or without varus/valgus component

**DOI:** 10.1097/MD.0000000000028337

**Published:** 2021-12-23

**Authors:** Xiao Zhang, Xiaochen Tian, Shuai Wang, Yaning Hu, Shuo Pan, Aqin Peng

**Affiliations:** a Department of Orthopedic Surgery, Third Hospital of Hebei Medical University, No. 139 Ziqiang Road, Shijiazhuang, Hebei, China; b Department of Orthopedic Surgery, People's Hospital of Shijiazhuang, No.36 Fanxi Road, Shijiazhuang, Hebei, China; c Department of Orthopedic Surgery, Hebei Chest Hospital, No. 372 Shengli Road, Shijiazhuang, Hebei, China.

**Keywords:** classification, CT, hyperextension tibial plateau fracture, morphology

## Abstract

This study aims to introduce a morphological classification of hyperextension tibial plateau fractures based on CT scans and to reveal the correlation between the anterior compression and posterior tension fractures.

From January 2015 to January 2019, 37 patients with hyperextension tibial plateau fractures were studied retrospectively. Based on this classification, the fractures were divided into 2 groups: group A had anterolateral or anteromedial compression fractures while group B had both. Three observers classified the fractures and recorded the morphology and incidences of posterior plateau fractures and proximal fibular fractures.

All 37 fractures were allocated to group A (n = 15; 40%) and B (n = 22; 60%). Of the posterior tibial plateau fractures, 10 (27%) fractures were defined as partial and 27 (73%) as total. Of the 37 fractures, 18 (49%) proximal fibular avulsion fractures were observed. There was a significant difference between groups A and B regarding the incidence of total posterior tibial plateau fractures (*P* < .05). However, there was no significant difference between the incidence of proximal fibular avulsion fractures in the 2 groups or the combined and non-combined type fractures in group B (*P* > .05).

Hyperextension tibial plateau fractures with a decreased posterior slope angle always involve both the anteromedial and anterolateral plateaus. This CT-based classification may improve the understanding of fracture features and is helpful for planning treatment.

## Abstract 2

Supplemental Digital Content is available in the text

## 1 Introduction

Of all the tibial plateau fractures (TPFs), hyperextension TPFs are a relatively rare subtype (prevalence 3%) and are often combined with multiple soft tissue injuries.^[1,2]^ It is simple and reliable to classify hyperextension TPFs according to coronal deformities, but *varus* or *valgus* type fractures contain several different subtypes that are not suitable for homogeneous comparison. Moreover, coronal deformity is evaluated through anteroposterior radiographs, inhibiting the detailed analysis of fracture features on the joint surface of the tibial plateau. Hence, a comprehensive, detailed hyperextension TPF classification based on CT is urgently needed.

In 2001, Chiba et al^[3]^ first showed the fracture pattern caused by hyperextension *varus* injury and that these fractures have a high incidence of associated ligament injuries. Bennett et al^[4]^ and Conesa et al^[5]^ described the formative mechanism of anterior small and large type compression fractures, respectively. These studies focused more on the relationship between anteromedial compression fractures and posterolateral ligament injuries but did not refer to concurrent posterior TPFs. Bicondylar *varus* hyperextension TPFs were first depicted by Firoozabadi et al,^[6]^ who summarized the radiographic hallmarks of this fracture. Gonzalez et al^[2]^ further expanded bicondylar TPFs into pure, *varus* and *valgus* types. However, “bicondylar” merely demonstrates that medial and lateral columns are involved and cannot show accurate fracture locations in anteroposterior direction. Few studies referred to the fracture morphology of posterior plateaus at all or briefly mentioned it as “simple.”^[6]^ Meanwhile, being an important risk factor for accelerated knee osteoarthritis,^[7]^ it is rational to classify the fractures based on coronal deformities, but rotational force is another issue requiring consideration.^[8]^ Currently, because of a lack of a commonly accepted classification system based on anatomic fracture morphology, some confusing terms are used to indicate fracture subtypes caused by hyperextension injuries, such as “reverse Segond” fractures^[9]^ and non-dislocated hyperextension TPFs.^[10]^

The purpose of this retrospective study, therefore, is to introduce a new systematic classification for hyperextension TPFs based on CT images and to illustrate the correlation between anterior compression fractures, posterior tension fractures and proximal fibular avulsion fractures (PFAFs).

## 2 Materials and methods

Ethical approval was obtained by the ethics committee of the Third Hospital of Hebei Medical University. All procedures were performed in accordance with the tenets of the Declaration of Helsinki.^[11]^ All patients gave their informed consent prior to their inclusion in the study.

From January 2015 to January 2019, a total of 37 patients (25 male and 12 female) with unilateral hyperextension TPF were selected from 543 potential TPF patients. The mean age of the patient was 51.0 ± 8.9, with a range from 23 to 63 years old. The causes of the injuries included falling from a height (n = 14), vehicle accident (n = 13), motorbike or electric motorbike accident (n = 7), and sports-related injuries (n = 3).

Diagnosis was established based on the previously described definition by Gonzalez et al^[2]^

1. anteromedial or/and anterolateral compression fractures,
2. tension failure of partial or total posterior cortex, or proximal fibular avulsion fractures,
3. posterior tibial slope angle (PTSA) less than 11°.^[12]^

The inclusion criteria were patients aged between 18 and 65 years with a complete collection of radiographs and CT images. The exclusion criteria included:

1. patients aged below 18 years because of their immature bones,
2. patients older than 65 because of the possible existence of osteoporosis,
3. pathologic fractures,
4. early fractures or knee arthritis with the abnormal alignment of lower extremities,
5. other fractures occurring on the same lower extremity such as femur and patella fractures.

### 2.1 Description of the new classification

**Figure 1:**
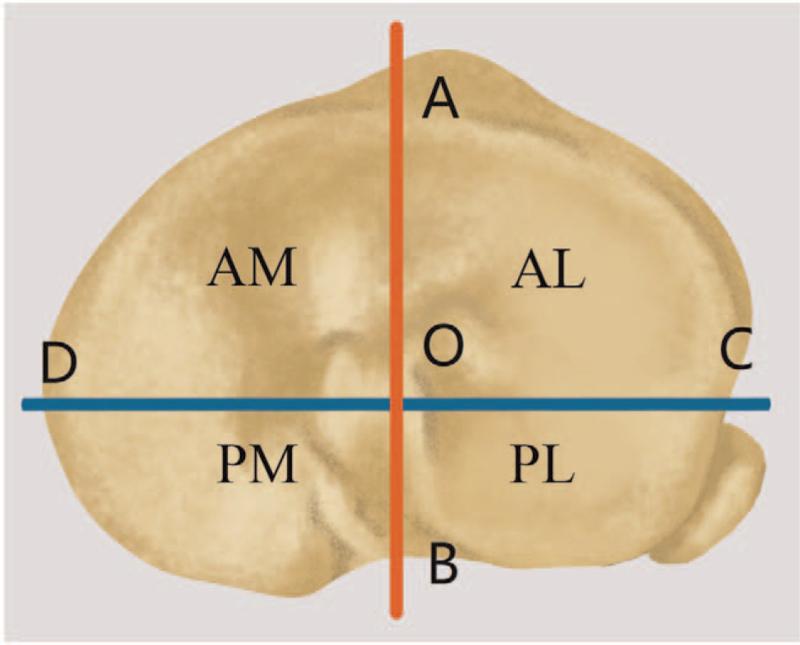
Illustration of 2 column and 4 quadrants of tibial plateau. (A) medial 1/3rd of tibial tuberosity; (B) posterior sulcus; (C) the anterior boarder of fibular head; (D) posteromedial ridge of proximal tibial; O, middle point of tibial eminences; AM = anteromedial quadrant, AL = anterolateral quadrant, PM = posteromedial quadrant, PL = posterolateral quadrant.

In order to illustrate the new classification better, each tibial plateau was divided into medial and lateral columns. The medial column was further divided into anteromedial and posteromedial quadrants, and the lateral column was divided into anterolateral and posterolateral quadrants according to Anwar et al^[13]^ study (Fig. 1).

**Figure 2:**
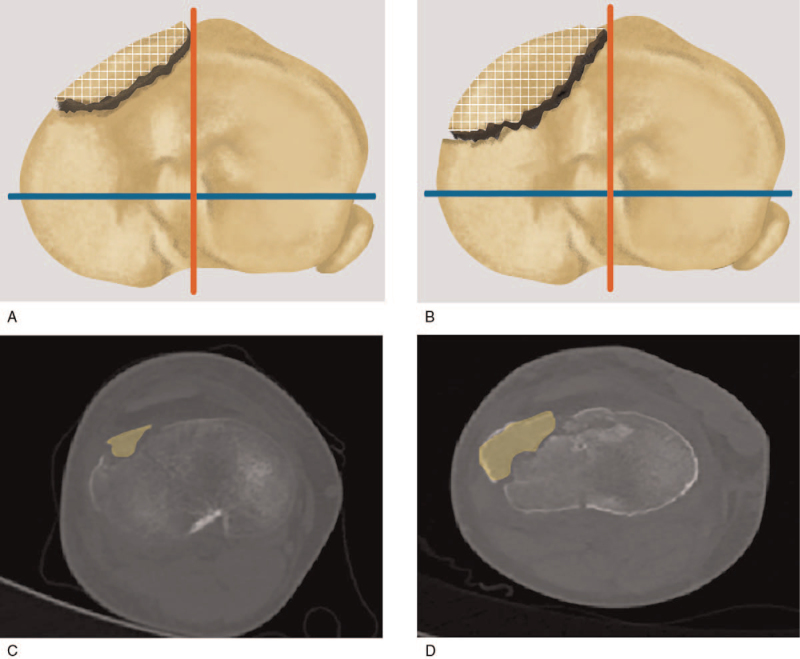
Small and large type fractures (grid districts) are divided by the ratio of the length of anterior fragments and corresponding column in the anteroposterior direction. (A) Small type: a quarter or less than the medial or lateral column. (B). Large type: more than 1/4 of the column. (C, D). Sample graphs of the small type and large type fractures (yellow districts).

**Figure 3:**
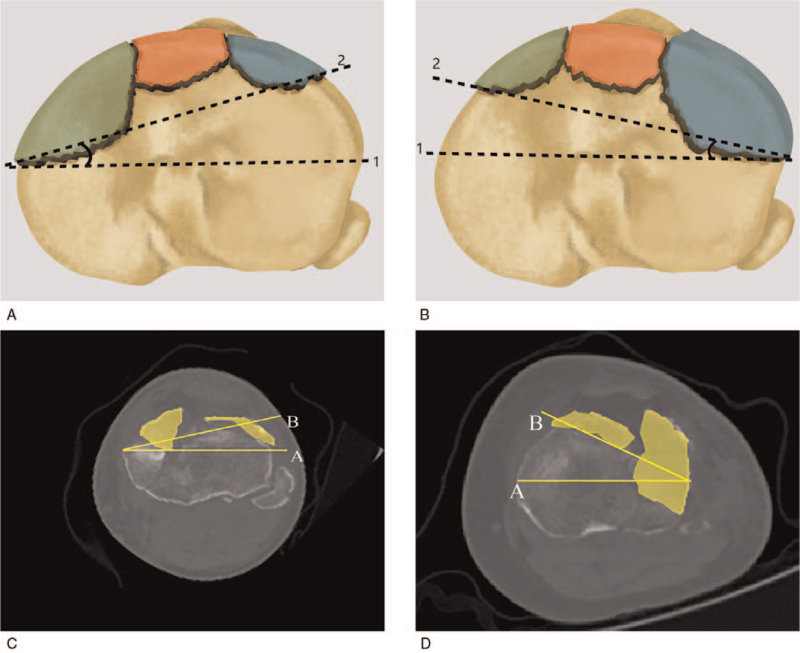
Internal and external rotation of the combined type fractures. Line A is the maximal diameter of tibial plateau, and line B links the posterior margin of anterior fractures (black dote lines). The angle created by line A and B predicts the knee rotation. (A) The angle points to medial, representing internal rotation; (B) The angle points to lateral, representing external rotation; (C, D), Sample graphs of internal and external rotation (the angle created by yellow lines).

**Figure 4:**
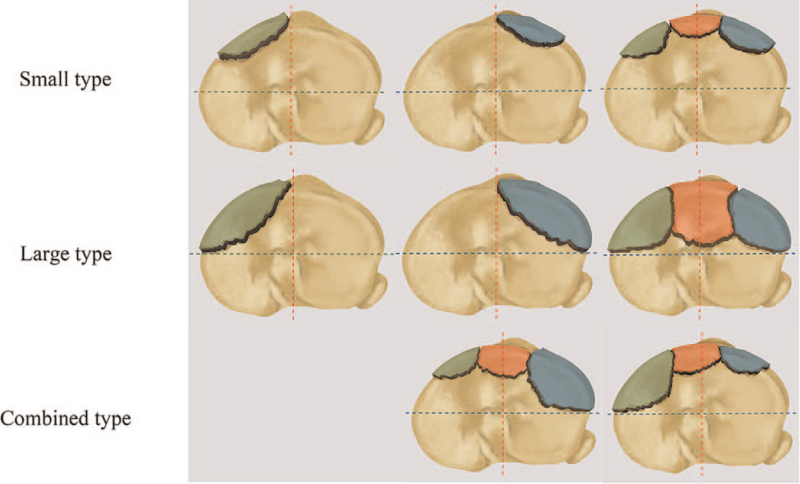
Illustrations of the proposed classification. Green, blue and red districts represent anteromedial, anterolateral and anteriormedian fragments, respectively. The size of anterior colorful districts represents the small or large type fractures.

Our classification is comprised of 2 groups. Anterolateral or anteromedial compression fractures are allocated to group A while fractures involving both anterior (anterolateral and anteromedial) quadrants including anteromedian plateau (segment d in Yao et al^[14]^ study) are allocated to group B. Chiba et al^[3]^ divide the anterolateral or anteromedial compression fractures into a small or large type based on the fragment size (Fig. 2). The combined fracture type is defined as having both a small and a large anterior fragment, and it shows the knee was rotating at the time of injury (Fig. 3). The final classification of diagrammatic drawing is shown in Figure 4. All the demographic and classified information are showed in Table 1.

**Table 1:** Baseline demographic and clinical characteristics for 37 patients.

Age (Mean ± SD, yr)	51.04 ± 8.92
Gender (female vs male)	12 vs 25
Side (left vs right)	14 vs 23
Injury mechanism (n)	
Falling from a height	14
Vehicle accident	13
Motorbike accident	7
Sports-related injury	3
Schartzker classification	
IV	6
V	19
VI	12
Posterior slope angle (Mean ± SD, Range, °)	0.78 ± 6.19, −21–9
New classification	
Group A (n)	15
Age (Mean ± SD, yr)	49.87 ± 9.60
Side (left vs right)	4 vs 11
Posterior slope angle (Mean ± SD, Range, °)	0.93 ± 5.70, −10–9
Group B (n)	22
Age (Mean ± SD, yr)	51.86 ± 8.54
Side (left vs right)	10 vs 12
Posterior slope angle (Mean ± SD, Range, °)	0.68 ± 6.64, −21–9

**Figure 5:**
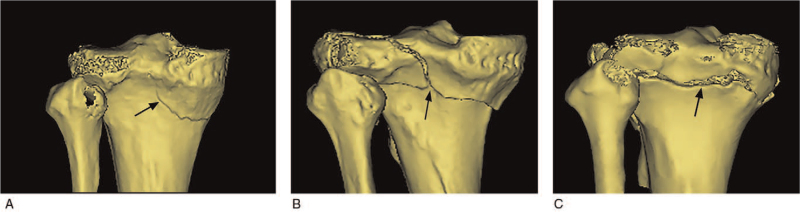
Posterior tibial plateau fractures are divided based on the scale of the fragments (black arrows) departed from tibial metaphysis. Total type includes separated and intact subtypes. (A). Partial type. (B). Separated subtype. (C). Intact subtype.

X-rays and CT scans were obtained using the Picture Archive and Communication System database (PACS). 3D reconstructive images were reconstructed using MIMICS software (Version 20.0, Materialise, Leuven, Belgium). In order to visualize the articular surface of the reconstructed tibial plateau, we removed the femur and patella. All the radiographs, CT scans and 3D images of the selected fractures were reviewed by 3 senior orthopaedic surgeons. They first independently evaluated the images and then worked together to reach a consensus about the new classification, and all of the fractures were classified. Then, they observed the morphological characteristics of posterior TPFs. According to the size of fragments lost from the posterior tibial metaphysis, the posterior TPFs were allocated as partial or total, and then they were further divided into separated and intact subtypes (Fig. 5). The incidences of posterior fracture types and subtypes were recorded, as well as the incidence of PFAFs. The illustration of the new classification and related fracture data are given in the Table 2.

**Table 2:** Illustration of the new classification and the incidences of related fractures.

Group	Type	Location	Number	PMQs	PLQs	IPQs	IMQs	PFAFs
A	S	Al	1	–	1	–	1	–
		Am	4	2	–	–	–	3
	L	Al	7	–	5	–	6	–
		Am	3	1	1	–	–	2
B	S		8	1	2	5	–	4
	Combined	An	11	9	9	2	–	9
	L		3	3	3	–	–	–

Al = anterolateral quadrant, Am = anteromedial quadrant, An = anterolateral and anteromedial quadrants, PMQs = posteromedial quadrants, IPQs = intact posterior quadrants, IMQs = intact medial quadrants, L = large type, PLQs = posterolateral quadrants, PFAFs = proximal fibular avulsion fractures, S = small type.

### 2.2 Statistical analysis

The categorical data were compared with two-tailed Fisher exact tests to assess the correlation between anterior and posterior TPFs, as well as PFAFs. Analyses were performed using SPSSfor windows statistical software (ver. 18.0; SPSS Inc., Chicago, IL). A *P* value of less than .05 was considered statistically significant.

## 3 Results

Based on our new classification, all 37 fractures were allocated to group A (n = 15; 40%) or group B (n = 22; 60%). Of the 37 fractures, 10 (27%) were defined as partial and 27 (73%) were defined as total. In our series, PFAFs were found in 18 (49%) fractures.

In group A, there were partial (n = 10) and total (n = 5) fractures (separated subtype, n = 5; intact subtype, n = 0). In the large type of group A, 6 out of 7 fractures involved anterolateral quadrants associated with intact medial column fractures.

In group B, there were partial (n = 2) and total (n = 20) fractures (separated subtype, n = 13; intact subtype, n = 7). Of the 22 group B fractures, 11 were categorized as combined fractures, and of these, PFAFs were found in 9 (82%) cases.

There was a significant difference between groups A and B regarding the incidence of total posterior TPFs (*P* = .006). Regarding the incidence of PFAFs, there was no significant difference between group A and B (*P* = .092) (Table 3), and also between combined and non-combined type fractures in group B (*P* = .08).

**Table 3:** Illustration of the relationship between anterior and posterior fractures.

Group	PFAFs	Total posterior fractures	
		Separated	Intact
A	6	5	0
B	13	13	7
*P* value	.092		.06

PFAFs = proximal fibular avulsion fractures.

## 4 Discussion

This new CT-based classification of hyperextension TPFs clarifies the relationship between the subtypes and the incidence of related fractures. We found that the hyperextension TPFs with a decreased PTSA which involved anterior quadrants were common and often associated with total posterior fractures. Meanwhile, the small type of anterior compression fractures tended to be found with intact posterior TPFs, and the large type of anterolateral compression fractures was always connected with intact medial column fractures.

Utilizing CT scans has opened a new chapter in TPF classification. Chan et al^[15]^ found the agreement with the pre-operation plan increased from 58% to 71% with an additional CT scan which enhanced the reliability of different classifications,^[16]^ but whether 3D-CT can improve the reliability of characterization and classification of TPFs is still debatable.^[17,18]^

In a comparative study including 30 knees, Hirschmann et al^[19]^ found 3D-CT could minimize measurement deviations because it could overcome the influence of the orientation of the patient's legs at the time of the CT scan and the width of the CT slices. By mesuring 10-case 3D reconstructed TPF models, Assink et al^[20]^ concluded that 3D-CT could show the exact fracture characteristics such as step-off and displacemet of articular fractures. Therefore, 3D-CT reconstruction has advantages over ordianary CT scans, and it was applied in this study.

Concern has been given to injury mechanisms and related soft tissue injuries. Through a retrospective analysis of 514 TPFs, Hua et al^[1]^ found only 16 fractures abided by the diagnostic criteria of hyperextension TPFs, and up to 13 fractures involved both anterior quadrants. Of these 13 fractures, it was observed that all of the posterior tension TPFs were intact, contrasting with our observations. Xie et al^[21]^ adopted a 3D fracture mapping technique to analyze the correlation between injury mechanism and fracture morphology and found that the *varus* and *valgus* hyperextension TPFs constituted 13% and 3% of the 353 TPFs in their case series, respectively. Of the 47 *varus* fractures, 24 fractures were combined with PFAFs and the size of medial plateau fragments was varied. Luo et al^[22]^ innovatively viewed the posterior tibial plateau as a single column. In a case series of 74 bicondylar TPFs, Streubel et al^[23]^ found 68% of the fractures had an average of over 5°difference of PTSAs between posterolateral and posteromedial fragments and concluded that ignoring the posterior TPFs might lead to inappropriate utilization of treatment techniques and poor outcomes.^[24]^

Subtypes in our classification showed a close relationship with injury mechanism, which can help us to understand fracture charcteristics. Partial posterior TPFs were often related to group A fractures, which might often caused by low-energy trauma such as sport-related injury, and *varus* or *valgus* force might be the main factor^[25]^; while total posterior TPFs were always associated with group B fractures that were highly likely to result from high-energy trauma, and hyperextension force plays an more important role than *varus* or *valgus* force, leading to relatively more severe posterior fractures. In addition, based on the correlation between intact posterior fractures and small type fractures in Group B, this fracture pattern may be caused by an axial force on the over-hyperextension knee, and further leaded to both the intact posterior shear fractures and anterior compressed fractures quadrants, or servere posterior soft tissue injury, such as posterior capsule disruption, anterior and posterior cruciate ligament injuries, without definite posterior fractures.^[26]^ If a fracture cannot be classified into any type of our classification in clinical practice, extra attention should be paid for latent soft tissue injury. Therefore, a reasonable fixation process can be made due to the principal reduction and fixation through the converse injury mechanism based on this classification in clinical practice.

Many studies^[8,14,27]^ have proposed the hypothesis that knee rotation might play a role in hyperextension TPFs, but no study has yet proved it with concrete evidence. According to our classification, the combined type fractures can indicate knee rotation because the different sizes of anterolateral and anteromedial fragments show the relative location between the femur and tibia when an injury occurs. Regarding the concurrent PFAFs, there was no significant difference between combined type fractures and other type fractures in group B, which means hyperextension force might play a more important role than rotation force. However, the combined type fractures had a high incidence of concurrent PFAFs (82%), which is likely to have resulted from both knee rotation and hyperextension forces during the injury process because of the rotation-resisting and hyperextension-resisting function of ligaments attached to the proximal part of the fibula. Therefore, it is necessary to fix the concurrent PFAFs or lateral collateral ligaments if the injured knee instability still exists after tibial fracture fixation (Supplemental Digital Content Figure S1, http://links.lww.com/MD/G546).

The advantages of our classification include: first, it is a comprehensive and detailed classification based on the fracture morphology of 4 quadrants of tibial plateau. This anatomically oriented classification including 2 columns and 4 quadrants can be easily remembered and used, and previous literature^[13]^ showed good intraobserver and interobserver reliability, the mean Kappa value was 0.939 and 0.955, respectively. Second, it may facilitate hyperextensive TPF research because it contains all of the hyperextension TPF patterns reported in previous literature^[2,3,6,10,23]^ and each fracture pattern only matches 1 certain type. Finally, it helps us choose suitable operative approaches for fracture fixation. Before fixing anterior fragments, the reduction of posterior TPFs should be considered first, and an additional posterior approach may be needed for separated posterior TPFs.^[28]^ Regarding group A fractures, 1 anterolateral or anteromedial surgical exposure is straightforward based on the location of anterior fragments, but except for those little posterior fragaments with a stable knee joint, another posterior incision is always necessary in the diagonal direction of the anterior fractures. Anteromedial compression fractures in group A often combine with PFAFs, which need to be fixed through an additional lateral approach; generally, the anterolateral and medial incisions are adopted when anterolateral compression fractures combined with intact medial plateau fractures (Supplemental Digital Content Figure S2, http://links.lww.com/MD/G546). Moreover, this fracture pattern should be concerning because they resemble Wahlquist type C fractures, which have a high possibility of severe concurrent complications, such as compartment syndrome and neurovascular injures.^[29]^ Regarding the majority of group B fractures, the anterolateral and posteromedial approaches are recommended^[6,30]^ (Supplemental Digital Content Figure S3, http://links.lww.com/MD/G546), but for small type fractures of group B, the modified anterior middle approach is suggested.^[31]^

However, our classification has several weaknesses, one of which is that the anteromedial or/and anterolateral compression fractures without posterior TPFs or PFAFs, potentially associated with multiple ligament injuries, were not selected in our study. These are, however, also created by hyperextension injuries. In light of this, an MRI scan is necessary for evaluating the related ligament injuries. The second weakness is how to accurately and quantitatively measure the knee rotation angles of different types in our classification. The authors suggest conducting a large multi-center study to overcome these drawbacks.

The limitations of the study are as follows. First, the statistical sample size was small, which reduced the validity of the study and increased the margin of error. Second, the inherent features of the retrospective study, as well as the observers’ preference, experience, and ability may have influenced the classification of the fractures. Third, ligament injuries may also affect the clinical outcomes, but assessing these injuries was impossible because MRI was not performed in this study.

## 5 Conclusion

The hyperextension TPFs with a decreased PTSA tend to involve both anterior quadrants which are frequently combined with total posterior TPFs. Our classification focuses on the morphological characteristics of and correlation between anterior compression fractures and related posterior tension fractures, which may enhance our understanding of hyperextension TPFs and help us make more accurate pre-operative planning.

## Acknowledgments

Grateful acknowledgement is made to my three colleagues Professor Xu Zhang, Xinzhong Shao, and Yadong Yu who voluntarily classified the selected fractures using their deep clinical experience and precious time. We also sincerely appreciate Tom Speed who helped us to proofread the article.

## Author contributions

**Conceptualization:** Aqin Peng.

**Data curation:** Xiao Zhang, Xiaochen Tian.

**Formal analysis:** Shuai Wang, Yaning Hu, Shuo Pan.

**Investigation:** Xiaochen Tian, Shuo Pan.

**Project administration:** Xiao Zhang.

**Resources:** Xiaochen Tian.

**Software:** Shuai Wang, Yaning Hu.

**Supervision:** Aqin Peng.

**Writing – original draft:** Xiao Zhang.

**Writing – review & editing:** Xiao Zhang.
